# Diffuse Aspiration Bronchiolitis With Neuromyelitis Optica Spectrum Disorder

**DOI:** 10.7759/cureus.38429

**Published:** 2023-05-02

**Authors:** Keisuke Mine, Hideki Mori

**Affiliations:** 1 Department of Respiratory Medicine, Second Department of Internal Medicine, Nagasaki University, Nagasaki, JPN; 2 General Internal Medicine, National Hospital Organization Nagasaki Medical Center, Omura, JPN

**Keywords:** case report, neurological diseases, aspiration, neuromyelitis optic spectrum disorder, diffuse aspiration bronchiolitis

## Abstract

Diffuse aspiration bronchiolitis (DAB) is a chronic inflammatory response of the bronchioles caused by repeated aspiration of foreign bodies. It is common among older individuals with dysphagia associated with neurological diseases or dementia. Here, we present the case of a woman in her 40s who was presumed to have developed DAB due to neuromyelitis optica spectrum disorder (NMOSD). There have been no reports of DAB due to NMOSD. The absence of obvious episodes of aspiration and the fact that pneumonia was the predominant symptom delayed the diagnosis despite the appearance of specific neurological abnormalities. DAB caused by neurological diseases of the brainstem should be considered in younger patients with diffuse centrilobular opacities, even if dysphagia is not obvious.

## Introduction

Pulmonary diseases caused by aspiration are classified into the following three categories: chemical pneumonitis caused by aspiration of gastric acid, bacterial aspiration pneumonia mainly caused by bacterial infection due to aspiration, and mechanical obstruction [1]. Some conditions are characterized by chronic bronchial involvement, with gross and histologic findings resembling diffuse panbronchiolitis, which Matsuse et al. described as diffuse aspiration bronchiolitis (DAB) [2]. DAB is thought to be caused by the aspiration of relatively small amounts of food or oral contents into the airway. It may occur in patients with underlying conditions that predispose them to aspiration, such as gastroesophageal reflux disease, cerebrovascular disease, and neuromuscular disease. Although occurrences in younger patients have been reported, it can be challenging to suspect the pathogenesis of aspiration in these individuals, which may lead to a delayed diagnosis [3-5]. In this report, we present the case of a young woman with DAB associated with neuromyelitis optica spectrum disorder (NMOSD).

## Case presentation

**Figure 1:**
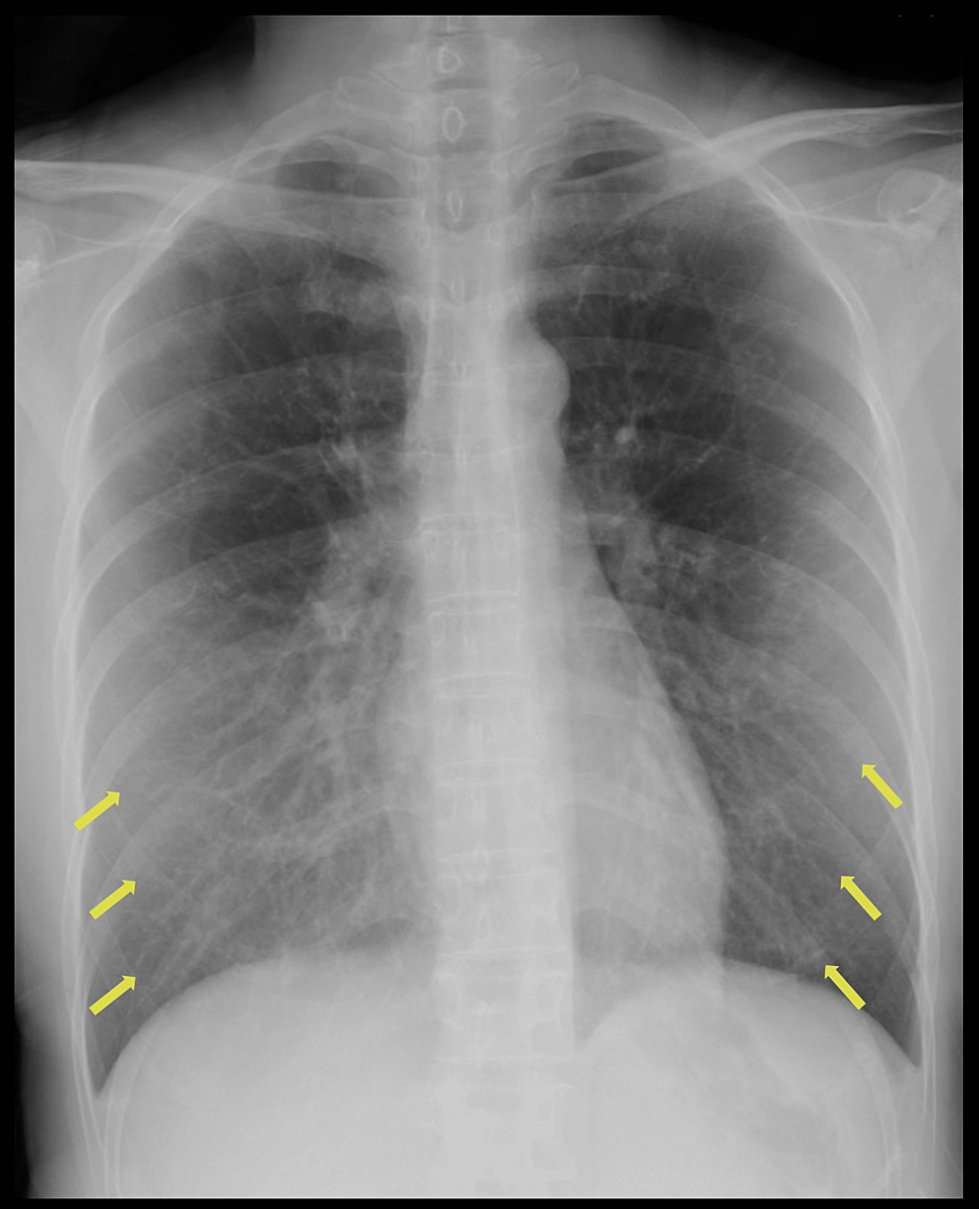
Chest radiographs showing frosted and granular shadows predominantly in the bilateral lower lung fields.

**Figure 2:**
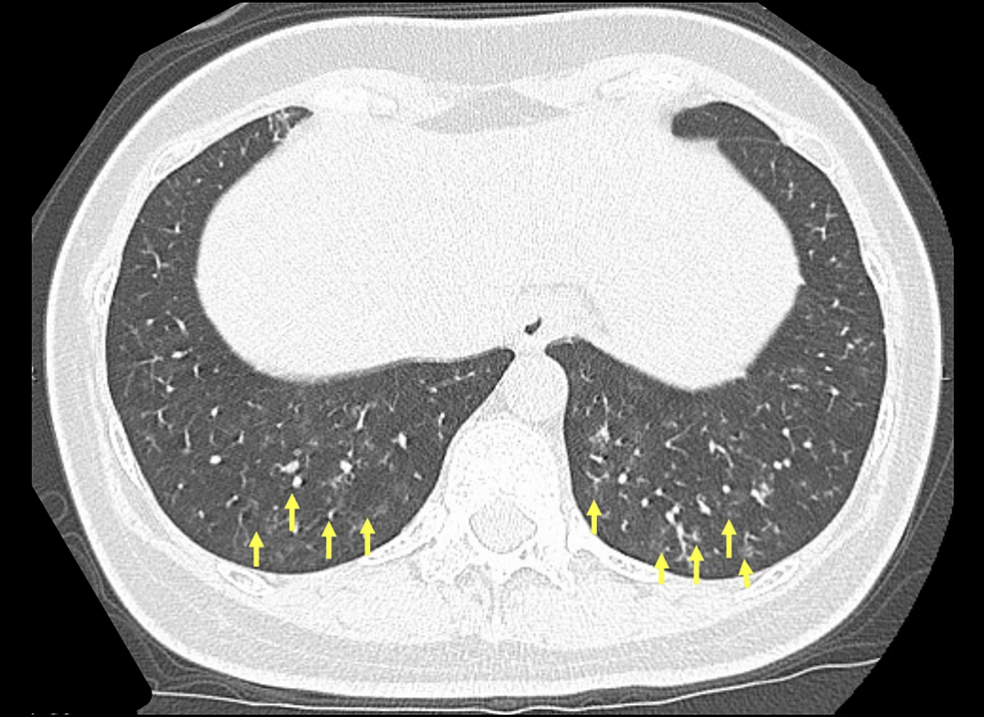
Chest computed tomography showing bilateral diffuse lobular central pale granular shadows.

A 45-year-old female who underwent uterine fibroid surgery visited our hospital with a fever, productive cough, and dyspnea. The patient had no known past medical history except uterine fibroid status post-recent enucleation about 1.5 months before presenting to the hospital. She had been experiencing nausea and hiccups even before the surgery. Her postoperative course was unremarkable. She was discharged on postoperative day seven. Although her nausea and hiccups had temporarily improved, they continued to persist. However, fever and productive cough appeared five days before the hospital visit. She visited a nearby primary care physician because her symptoms did not improve after self-monitoring. She was suspected of having pneumonia. Vital signs at the time of the hospital visit included the following: blood pressure, 120/71 mmHg; heart rate, 85 beats/minute; respiratory rate, 24 breaths/minute; oxygen saturation, 92% (3L/minute O_2_), and body temperature, 38.4°C. The superficial cervical lymph nodes were not palpable, and no goiter or heart murmur was noted. There were no adventitious sounds upon auscultation of either lung, nor were there any abdominal abnormalities, rashes, cyanosis, edema, joint deformation, or swelling. Chest X-ray performed in the sitting position (Figure 1) revealed bilateral reticular opacities in the lower lung fields. Chest computed tomography at the first visit (Figure 2) showed diffuse centrilobular nodular opacities and signs of bilateral bronchiolar dilatation in the lungs. Laboratory investigations revealed an elevated C-reactive protein level (31.98 mg/dL). Gram stain and sputum culture showed oral bacteria.

**Figure 3:**
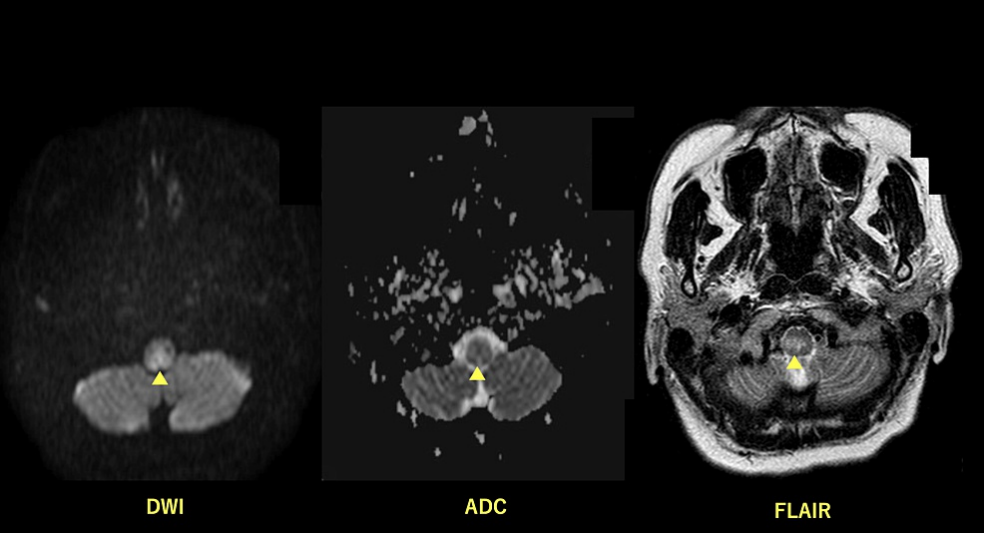
Brain magnetic resonance imaging scan diffusion-coordinated image showing a high-intensity area in the medulla oblongata. No ADC reduction was observed, and T2WI and FLAIR showed high intensity in the same area. DWI: diffusion-weighted image; ADC: apparent diffusion coefficient; T2WI: T2-weighted imaging; FLAIR: fluid-attenuated inversion recovery

The patient was administered ceftriaxone (2 g/day) and azithromycin (500 mg/day) for community-acquired pneumonia in younger individuals. Her fever rapidly subsided, and inflammatory markers decreased once antibiotic treatment was initiated. However, nausea, sensory disturbance affecting all four limbs, trunk ataxia, and spontaneous vertical nystagmus appeared on hospital day four. Diffusion-weighted, T2-weighted, fluid-attenuated inversion recovery imaging of brain magnetic resonance imaging revealed a high intensity in the medulla oblongata (Figure 3). Fiberoptic endoscopy revealed decreased swallowing function, aspiration of a large amount of saliva, and signs of left vocal cord paresis. The diagnostic criterion for NMOSD with a positive anti-aquaporin-4 (AQP4) is at least one core clinical characteristic. The patient was diagnosed with NMOSD based on a positive AQP4 antibody test and acute brainstem syndrome, one of the core clinical characteristics. The dysphagia gradually improved after five courses of steroid treatment with methylprednisolone (500 mg/day). The patient visited the outpatient department for immunosuppressive therapy and rehabilitation to prevent relapse. We initially suspected general community-acquired pneumonia and did not consider DAB as a differential diagnosis because the radiological findings suggested that pneumonia developed in a younger individual during an acute course. The patient responded to some extent but had a persistent wet cough and other symptoms. Although the patient had a pathophysiological involvement in bacterial infection, which may have led to responses such as fever and improvement in inflammatory markers levels, the aspiration was persistent because of persistent dysphagia caused by the underlying brainstem lesion. Other imaging findings, such as diffuse shadows, led to an overall diagnosis of DAB. Neurological abnormalities, including dizziness, sensory disturbances in all four limbs, ataxia, and spontaneous vertical nystagmus, were observed, although respiratory symptoms were temporarily alleviated by antibiotic treatment. A detailed examination of the swallowing function revealed dysphagia, and the patient was diagnosed with DAB caused by brainstem lesions due to NMOSD based on abnormal imaging findings in the brainstem and a positive anti-AQP4 antibody test.

## Discussion

The differential diagnosis of branching diffuse centrilobular nodular opacities includes infectious diseases such as viral pneumonia, mycoplasma pneumonia, pulmonary mycobacteriosis, chronic sinobronchial syndrome (diffuse panbronchiolitis), human T-cell lymphotropic virus type 1-associated airway lesions, pulmonary tumor thrombotic microangiopathy, and DAB. DAB is common in older individuals with dysphagia associated with neurological disease or dementia. However, DAB has been reported to develop as a complication of esophageal achalasia even in younger individuals [3-6]. Characteristic signs of NMOSD include dysphagia and hiccups due to brainstem lesions, eye movement disorders, impaired hearing, facial palsy, trigeminal neuropathy, dizziness, and ataxia [7]. To our knowledge, there have been no previous reports of NMOSD complicated by DAB.

The patient did not show any obvious signs of dysphagia. Retrospectively, the first symptom of NMOSD in the present case is believed to have been hiccups noted 1.5 months before gynecological surgery; however, we did not initially suspect a relationship between hiccups and the abnormal pulmonary opacities observed during the first visit because the symptom had temporarily improved. It is not uncommon for this to be overlooked or to require a fair amount of time to diagnose DAB because obvious aspiration episodes may be missed in many cases.

This is a case report of DAB secondary to NMOSD. Although there have been reports of DAB caused by brainstem lesions such as brain tumors, there have been no reports of NMOSD, making this case novel. It is clinically crucial because the pathophysiology of DAB itself is often overlooked or time-consuming owing to the lack of obvious aspiration episodes, and the diagnosis of a potentially early-treatable condition such as NMOSD is often delayed.

## Conclusions

DAB with brainstem involvement should be considered a differential diagnosis in the presence of diffuse centrifugal nodular opacities, even in relatively young patients with an acute history. A high index of suspicion is critical because dysphagia is not always obvious and specific symptoms of the brainstem may appear late.
